# Interventional Analgesic Management of Lung Cancer Pain

**DOI:** 10.3389/fonc.2017.00017

**Published:** 2017-02-14

**Authors:** Uri Hochberg, Maria Francisca Elgueta, Jordi Perez

**Affiliations:** ^1^Cancer Pain Program, McGill University Health Centre, Montreal, QC, Canada; ^2^Alan Edwards Pain Management Unit, McGill University Health Centre, Montreal, QC, Canada

**Keywords:** lung neoplasms, cancer pain, nerve block, spinal anesthesia, cementoplasty, neurostimulation

## Abstract

Lung cancer is one of the four most prevalent cancers worldwide. Comprehensive patient care includes not only adherence to clinical guidelines to control and when possible cure the disease but also appropriate symptom control. Pain is one of the most prevalent symptoms in patients diagnosed with lung cancer; it can arise from local invasion of chest structures or metastatic disease invading bones, nerves, or other anatomical structures potentially painful. Pain can also be a consequence of therapeutic approaches like surgery, chemotherapy, or radiotherapy. Conventional medical management of cancer pain includes prescription of opioids and coadjuvants at doses sufficient to control the symptoms without causing severe drug effects. When an adequate pharmacological medical management fails to provide satisfactory analgesia or when it causes limiting side effects, interventional cancer pain techniques may be considered. Interventional pain management is devoted to the use of invasive techniques such as joint injections, nerve blocks and/or neurolysis, neuromodulation, and cement augmentation techniques to provide diagnosis and treatment of pain syndromes resistant to conventional medical management. Advantages of interventional approaches include better analgesic outcomes without experiencing drug-related side effects and potential for opioid reduction thus avoiding central side effects. This review will describe various pain syndromes frequently described in lung cancer patients and those interventional techniques potentially indicated for those cases.

## Introduction

### Prevalence of Pain in Patients Diagnosed with Lung Cancer

Cancer and pain are clinical entities closely associated. Recent reviews suggest a prevalence of pain in cancer patients of 51% regardless of the type and stage. This prevalence increases with the type of tumor (head and neck, lung, and breast cancers are the ones with higher prevalence) and with the staging (advanced, metastatic, or terminal) reaching a 66% of cases (1).

Recent therapeutic advances have allowed increased survival rates potentially turning lung cancer into a chronic condition (2). Since pain is present in up to 39% of cases after curative intent, an increased survival could potentially impact this number of patients left with persistent symptoms despite being successfully treated.

### Importance of Appropriate Symptom Control in Lung Cancer Patients

Undertreated cancer pain associates both physical and psychological consequences, causing suffering and reduced quality of life. Patients with unrelieved pain associate physical symptoms like insomnia, anorexia, profound fatigue, reduced cognition, and overall reduced vital capacity. Cancer patients presenting with unalleviated pain withdraw from social and familial interactions leading to isolation and psychological distress. Lastly, persistent pain can cause existential and spiritual suffering, which can limit the patient’s coping skills (3).

### Basic Pharmacological Management to Relieve Cancer Pain

The World Health Organization (WHO) responded to an essential necessity to assess and treat cancer pain by designing in 1986 the WHO Cancer Pain Relief guidelines [updated 10 years later (4)]. Adoption to the three step ladder approach leads to satisfactory cancer pain control in the majority of cases. However, in a significant proportion of patients, appropriate conventional medical management following the WHO guidelines do not warrant satisfactory analgesic control or may provoke limiting drug-related side effects (5). For those cases, interventional cancer pain management may represent a valid option.

### Definition of Interventional Pain Medicine

Interventional pain management is a subspecialty of medicine devoted to the use of invasive techniques such as joint injections, nerve blocks and/or neurolysis, neuromodulation, and cement augmentation techniques to provide diagnosis and treatment of pain syndromes unresponsive to conventional medical management.

The basis of interventional pain practice lays on a profound knowledge of the anatomy and particularly the sensory innervation of different anatomical structures. When assessing a cancer pain case, aside from physiopathological considerations, the interventionalist may reflect about what is the anatomical structure that is hurting and which is the nerve supplying sensation to that structure.

As a principle, injections are avoided to be performed in the close vicinity of tumors for several reasons: (1) an increased risk of bleeding caused by abnormal tumor vascular neogenesis; (2) a risk of seeding cancer cells along the needle track, and (3) there is a risk of missing the target if the tumor has distorted the local anatomy. Routinely, nerve blocks are performed at levels where nerves are not damaged but are found proximal to the site of where the pain is coming from.

Several interventional cancer pain procedures have demonstrated effectiveness in relieving drug-resistant cancer pain symptoms (6), yet the evidence is scant. This may explain why interventional procedures have not yet been adopted in clinical guidelines for the management of cancer pain and thus remain optional to teams with trained clinicians on board.

### Scope of the Paper

This article aims to review the most common pain syndromes described in patients diagnosed with lung cancer. Treating physicians must be aware that conventional medical management is sufficient to achieve satisfactory pain management in most cases. Readers are encouraged to be familiar with comprehensive medical reviews on basic pharmacological analgesic approaches beyond the principles of the WHO (7, 8). The second part of this review lists the available interventional pain techniques indicated in cases of poor response to conventional medical management. A brief explanation of each technique with its peculiarities and scientific evidences, when available, is presented.

## Clinical Pain Syndromes in Patients with Lung Cancer

### Pain in the Chest

#### Chest Wall Pain

Chest wall pain is a severe and disabling symptom. Over a half of lung cancer patients suffer from chest pain at diagnosis (9). Pain is usually ipsilateral to the tumor site and is described as dull, aching, persistent, and poorly localized. Chest pain can be particularly severe and better identified if secondary to a rib metastasis or when the primary tumor involves the chest wall or pleura. The majority of patients suffering from non-small cell lung cancer with chest wall invasion suffer from chest pain (10).

#### Costopleural Syndrome

It refers to the severe refractory chest pain, often observed in patients diagnosed with pleural mesothelioma. It is caused by tumor invasion of the pleural cavity and thoracic wall, and it is seen often in the early stages of the disease. This chest pain can be pleuritic in nature and also described as a dull and poorly localized pain arising and involving part of the hemi-thorax. It normally develops during the course of the disease thus worsening with disease progression and often becoming challenging to relieve with conventional drug management. Generally, the pain will present with mixed nociceptive and neuropathic pain features as the autonomic, intercostal, and occasionally brachial plexus nervous structures are involved (11).

#### Rib Bone Metastases

The primary symptom resulting from bone inflammation is pain, which may have a pleuritic component when the parietal pleura is involved. Since lung cancer metastases to bone are predominately lytic, periosteal inflammation and breach is the most common mechanism of pain from bone metastases (12). Additionally, metastases to the ribs often come associated with intercostal nerve damage and thus neuropathic pain. The pain usually is localized in a particular area and is often reported at night or on weight bearing and with deep breathing. Pain is characteristically described as dull in character, constant in presentation, and progressively increasing in severity. At rest, the pain severity may be better controlled, thus patients may describe breakthrough pain related to postures and volitional or involuntary chest movements (13).

#### Pancoast Tumor

Pancoast tumor is defined as a malignant tumor arising from the lung apex, also referred to as superior sulcus tumor. The tumor usually affects adjacent structures such as ribs, blood vessels, and nerves (typically the lower nerve roots of the brachial plexus). As a result, patients may present with severe pain, often of neuropathic characteristics radiating toward the ipsilateral upper extremity and accompanied with sympathetic symptoms (like the Horner syndrome) caused by invasion of the cervicothoracic sympathetic ganglion. These manifestations often appear months prior to the diagnosis of the underlining disease (14).

#### Malignant Brachial Plexopathy

Tumor infiltration of the brachial plexus is commonly seen among patients with lung cancer. It usually affects the lower elements of the nervous plexus but at times it may evolve into a panplexopathy. Presenting symptoms are typically pain at the shoulder and upper extremity associating with weakness, muscle atrophy, and sensory deficits. As the tumor expands and invades adjacent structures, the likelihood of reaching the epidural space becomes substantial (15).

#### Post-Thoracotomy Pain Syndrome

Between 25 and 60% of patients undergoing thoracic surgery develop persistent postoperative pain following the procedure (16). Post-thoracotomy pain syndrome (PTPS) is defined as pain along the surgical bed lasting more than 2 months post-thoracic resection surgery (17). It may occur after thoracotomy for malignant or non-malignant lesions, it is usually restricted to one or more dermatomes. It is characterized by moderate to severe pain and typically described as numbness, tingling, burning, shooting, and sometimes itchy painful sensations. Sensory loss and allodynia are usually present as well. The exact mechanism for the pathogenesis of PTPS remains unclear and is probably a combination of neuropathic and myofascial pain (MFP) (18). Genetics, age, gender, preoperative stress, and perioperative pain have been identified as predisposition factors for PTPS. The type and extent of surgery are also factors for the development of chronic pain particularly when there is trauma to the intercostal nerves.

#### Postherpetic Neuralgia

Cancer patients increasingly suffer from acute herpetic neuralgia (19). The Varicella Zoster virus, which remains dormant at the dorsal root ganglion after primary infection, is reactivated under certain circumstances like aging and immunosuppression, causing a skin rash usually restricted to a dermatomal distribution. Upon resolution of the skin lesions, patients develop the commonly known as postherpetic neuralgia. This pain, neuropathic in nature (20), is found most frequently affecting thoracic dermatomes. Pain management for both acute and chronic forms is challenging and relies mostly on pharmacology-based approaches. In severe cases, when conservative treatment fails to provide satisfactory relief of postherpetic neuralgic pain, interventional approaches could be attempted (21).

### Bone Pain

Metastatic disease involving the musculoskeletal system is a common problem in oncology patients, occurring in up to 85% of patients diagnosed with breast, prostate, or lung cancer at the time of death (22). Bone metastases indicate a poor prognosis, with patients experiencing a median survival of 3 years or less; however, 5–40% of patients are alive at 5 years, dependent on tumor histology and disease burden (23). Metastatic bone disease leads to complications, such as pain, that can affect the patients’ quality of life. Bony metastases are frequent causes of pain among lung cancer patients as a result of pathological fractures, invasion of nearby pleural or visceral organs, involvement of neighbor nerve structures, spinal instability, and/or spinal cord compression. All of these complications are manifested as difficulty in ambulation or immobility and neurologic deficits (24). Pain symptoms arising from bone metastases present with mixed somatic and neuropathic features and are typically confined to a particular anatomical region. Pain often appears during the night and is exacerbated by weight bearing, posture change, or movement, thus with a strong dynamic component. Pain starts over weeks or months with progressive worsening becoming more severe and continuous at rest or with exacerbation triggered by dynamic changes (7). When baseline pain is well controlled but the patient experiences sudden and short lasting crisis of severe pain (also known as breakthrough pain), the case becomes more challenging since the crisis can be unpredictable and the available pharmacological options may be unsatisfactory.

Although thought to be the optimal management, surgery often cannot be offered because of underlying medical conditions, poor functional status, poor bone quality, or presence of multiple bone metastases (25). Currently, the gold standard symptomatic treatment of focal bone pain caused by metastatic disease is external beam radiation therapy. After radiation therapy most patients present with partial or complete pain relief; however, this relief is not achieved immediately but experienced after a considerable amount of time. In over 50% of patients, the pain relief is found temporary, and in 20–30% of cases the pain is not relieved (26, 27). Patients who underwent radiation therapy presenting with localized recurrent pain in the irradiated region are not usually candidates to receive more radiation because the potential toxic impact on non-cancer tissues.

Unfortunately, standard chemotherapy is often ineffective to treat metastatic-related pain. Bisphosphonates and denosumab are agents with proven benefits in decreasing severity of bone-related incidents in patients with metastatic bone disease. They may alleviate cancer-induced bone pain, yet there is insufficient evidence to recommend these therapies solely for pain relief purposes (28).

### Myofascial Pain

In approximately 10% of cancer patients, pain is unrelated directly to the disease or treatment and is most often originated in muscles and connective tissues (29). MFP is started to be recognized as one of the most important causes of pain in cancer patients during treatment, at terminal stages, or after curative therapy (30). MFP is a syndrome characterized by regional chronic pain associating multiple myofascial trigger points and fascial constrictions. It can appear in any body part and characteristically features focal point tenderness, reproduction of pain and hardening of the muscle upon trigger point palpation, pseudo-weakness of the involved muscle, referred pain, and restricted range of motion (31). The treatment for MFP includes physical therapies like myofascial trigger point needling and injections, myofascial release, and stretching exercises (32).

### Pain Related to Diagnostic Procedures and Cancer Treatment

Certain diagnostic test and treatments can cause or aggravate pain because they require the patient to maintain an immobile posture like imaging test or radiotherapy (RT). Others cause pain due to their invasiveness such as transthoracic needle biopsy or thoracocentesis (33). These acute pain episodes are described as transient exacerbations of pain typically well managed with conventional analgesic medications. Chemotherapy and RT are treatments frequently associated with deleterious and persistent painful syndromes that are not easily managed.

Chemotherapy-induced painful neuropathy is one of the most common and better studied pain syndromes consequences of cancer therapy. Most of chemotherapy-induced pains are self-limited and can be managed pharmacologically and/or with dose adjustments of the chemotherapeutic regimen. Probably, the better described chronic pain syndrome consequence of cancer treatment is chemotherapy-induced peripheral neuropathic pain (34).

Cancer patients can potentially suffer from RT-related pain both immediately after the treatment and as a late complication. During the acute phase, RT causes pain due to skin or mucosa inflammation or due to the procedure itself. Patients subject to RT for bone metastasis commonly suffer from pain flare-ups in radiated areas (35) and are treated usually with breakthrough analgesics and steroids. At later stages, RT-related pain can be caused by a variety of mechanisms including soft-tissue fibrosis and sclerosis and muscle weakness, such as thoracic pain, shoulder pain, and cervical dystonia (36). Thoracic cancer patients receiving greater dosages may require opioids to treat brachial plexopathy or chest wall pain following radiation (37, 38).

## Interventional Procedures

### General Principles of Interventional Pain Procedures in Cancer Patients

An interventional pain procedure is typically indicated when (a) the patient has not achieved satisfactory analgesic control despite optimal conventional medical management as suggested by the WHO guidelines or (b) when adequate pain control comes associated with intolerable side effects (39). Additional indications may include (c) favoring analgesic control with opioid sparing techniques or (d) analgesia in patients that are poor candidates to opioid analgesia.

The key to a successful partnership between treating oncologists and interventional pain physicians is communication to sharing the cases, reviewing indications and contraindications, appraising the available scientific evidences, and updating the team about the patient’s status and goals of treatment, in summary creating a clinical pathway for these patients.

Overall, interventional pain procedures should be offered to patients before they are too frail to undergo the procedure, thus they should not be considered an option in isolation but rather a part of an analgesic strategy. Patients should be able to consent and they should, along with their caregivers and the treating team, understand the procedure, the expected benefits and side effects, and potential complications (40).

A bidirectional communication between teams allows for earlier identification of candidates, thus preventing drug escalation and challenging cases. Additionally, the team must be updated on those fluctuations in the patient’s status that may potentially change the indication (risk of bleeding, infection, respiratory insufficiency). Following a successful procedure, the treating team must be vigilant for potential changes in analgesic requirements; ideally, opioids must be decreased to prevent central toxicity.

### Peripheral Nerve Injections

When cancer pain is experienced in the vicinity of an identified peripheral nerve, a temporary interruption of the pain transmission can be an effective method to control neuropathic pain. The term “nerve block” describes any procedure that utilizes a needle to deliver a local anesthetic or an ablative agent (phenol, alcohol, glycerin, etc.) for analgesic purposes. A block can have both diagnostic and therapeutic values. In order to identify the anatomical area and/or the afferent pathway involved in originating/conveying the pain sensation, a diagnostic nerve block may be effective. A prognostic block allows the decision to indicate a more complex and permanent procedure usually with neurolytic purposes. Diagnostic and prognostic blocks consist in injecting a small volume of a local anesthetic agent onto a nerve. The duration of the effect is usually short, depending on the potency of the local anesthetic agent injected. Patients are considered responders when most of their pain is significantly relieved during the following hours after the procedure. Neurolysis implies the focal destruction of nervous tissue as by the use of chemicals or thermal methods to disrupt nerve transmission. The classical targets for nerve blocks or neurolysis are sympathetic nerves or nerves with predominant sensory component. It is very important to always preserve motor and sphincter functions and when not possible, balance potential benefits against side effects before performing a neurolysis (40).

Among lung cancer pain patients, the most frequently targeted nerve structures are obviously located inside the thorax. As a general principle, the interruption of nociception must be attempted at a proximal site to the pain generator (41). Patients with thoracic chest wall pain may benefit from procedures targeting (from distal to proximal) the intercostal nerve, the posterior root of the thoracic radicular nerve, and the paravertebral space.

#### Intercostal Nerve Blocks and Neurolysis

It consists in injecting the neural structure located underneath each rib. This is a simple procedure that can be performed at the patient’s bed site not requiring advanced imaging guidance systems. Because the main complication is the pleural puncture and subsequent pneumothorax, it is suggested direct needle placement with ultrasonography. The injection of an intercostal nerve provides loss of sensation distal to the point of injection following the trajectory of the nerve toward the anterior chest wall. The largest series reporting intercostal nerve procedures for chest pain management include 25 patients with metastatic rib lesion undergoing intercostal blocks. In this study, 80% of the patients noted optimal local pain control and 56% experienced reduction in analgesic use after the procedure (42).

When a temporary intercostal nerve block provides adequate analgesia but limited to a short period of time, it may be reasonable to repeat the block adding a coadjuvant (43) or opting for a more permanent relief by damaging the nerve with a chemical neurolysis with phenol (44), a thermal neurolysis with heat using radio-frequency (RF) (45, 46) or freezing the nerve with cryoneurolysis (47).

#### Thoracic Nerve Root and Paravertebral Procedures

This consists of injecting the thoracic nerve roots at their exit from the spinal canal. These nerve roots can be injected individually (selective thoracic nerve block/neurolysis) or several at the same time by placing a needle at the thoracic paravertebral space. The selective nerve root block technique has been suggested as a proximal alternative site of injection in cases of post-thoracotomy pain (48). Authors described the use of pulsed RF, which delivers electricity to the dorsal root ganglion without causing nerve tissue damage. Results favored this technique over treatment of intercostal nerves and over conventional pharmacological management.

The injection of neurolytic agents into the thoracic paravertebral space presents advantages since one single injection may reach several thoracic nerve roots, thus involving a larger anatomical area. Neurolytic injection of the thoracic paravertebral space has been also described in cases of lung cancer with chest wall pain. In a small case series, injection of phenol in the vicinity of thoracic nerve roots provided satisfactory yet short lasting chest pain relief (49).

#### Brachial Plexus Procedures

Pain to the upper limb caused by lung cancer has been reported in cases of Pancoast tumors. The involvement of the sympathetic chain and the brachial plexus may cause neuropathic symptoms radiated toward the arm and the hand. Anesthetic techniques targeting the brachial plexus may include intermittent or continuous injection of local anesthetics (50) and neurolysis with phenol (51).

### Spinal Injections

Drugs injected into the spinal canal act through direct interaction with spine receptors thus achieving more potent analgesic effects with minimal doses. Additionally, the effect may be restricted to few dermatomes, hence sparing the possible side effects to a targeted anatomical area. The two modalities of intraspinal procedures available to manage drug-resistant pain secondary to lung cancer are continuous spinal drug delivery or spinal neurolytic procedures.

#### Continuous Drug Delivery

The basics of neuraxial analgesia consist of a catheter inserted into the spinal canal and a pump to administering medication in a continuous fashion. Opioids alone or combined with local anesthetics and other substances, such as clonidine or ziconotide, can be administered *via* epidural or intrathecal route to achieve neuraxial analgesia. Neuraxial analgesia allows the use of lower dosages of opioids, hence minimizing systemic side effects. As an example of the potency of intraspinal opioids: 300 mg PO morphine/day = 100 mg IV morphine/day = 10 mg epidural morphine/day = 1 mg intrathecal morphine/day (52).

Patient selection for spinal drug delivery includes choice of the anatomical space to deliver the drug (epidural vs. intrathecal) and choice of the administration mode (external infusion with syringe driver/pump vs. implanted reservoir with automated pump). The selection of the system is determined by factors like survival expectancy, body habit, patient admitted or ambulatory, financial resources, and/or expertise of the treating team (53).

For those patients with reduced life expectancy (<3 months), the neuraxial method of choice remains the epidural route. The main advantages of epidural opioid delivery are reduced risk of pharmacological complications, theoretical dermatomal analgesia achieved when combined with local anesthetics, decreased risk of post-dural puncture headache, and potentially, more familiarity within other specialties. On the other side, continuous epidural analgesia requires infusion of larger volumes of medication and a higher risk of catheter-related complications since it is not normally anchored or internally implanted (54).

Intrathecal drug delivery has been extensively described in the literature for the management of drug-resistant cancer pain syndromes. Available guidelines can be found to identifying the best candidates for this analgesic therapy (55). Advantages of intrathecal systems include better pain control with lower dosages, lower risk of catheter-related complications, and totally implanted systems thus, reduced rate of infections (56). Direct comparison of intrathecal drug delivery vs. conventional medical management favors the experimental arm in quality of analgesia, profile of side effects, and survival rates (57).

#### Intraspinal Neurolysis

Pain relief in terminal cancer cases achieved by means of injection of a neurolytic agent has been extensively reported (58). The key for a successful neurolytic procedure is balancing the expected analgesia and the potential nerve deficits associated.

These neurolytic procedures seem to be restricted to the latest option in the interventional cancer pain armamentarium (59) because they carry inevitable nerve deficits and because intraspinal drug delivery systems have become more available.

For lung cancer pain patients, the options include epidural (60) or intrathecal (58) injections of neurolytic agents such as alcohol or phenol. Because these neurolytic approaches are usually left as a last resort in the management of severe and drug-resistant cancer pain in terminally ill patients, the available evidences are only restricted to case series. From those evidences, it can be inferred that intraspinal neurolysis is a complex analgesic technique providing satisfactory analgesia but carrying a high potential for neurological deficits that must be weighted before performing the technique.

### Electrical Neuromodulation Techniques

Electrical neuromodulation is a technique by which an electrode that is placed next to a nervous structure stimulates selective small nerve fibers, which in turn inhibit nociception through complex physiological mechanism. Neurostimulation can be achieved *via* placement of electrodes under the skin (subcutaneous/field stimulation), close to peripheral nerves or to spinal nerve roots (peripheral nerve/DRG stimulation), inside the epidural space close to the ascending dorsal columns [spinal cord stimulation (SCS)], or inside the brain (deep brain stimulation). The efficacy, safety, and cost-effectiveness of neurostimulation techniques in the management of chronic pain of non-cancer origin have been sufficiently demonstrated in the last decade (61).

Conversely, because of its cost, the indication for neurostimulation in cancer pain patients is usually restricted to those cases when cancer has been successfully cured but patients are left with painful permanent consequences. There are no randomized trials addressing the benefits of SCS for cancer-related pain (62). Indications for SCS included chest wall pain (63) or chemotherapy-induced neuropathy (64), for example.

### Neurosurgical Procedures

Historically, destructive procedures for cancer pain were the main line of treatment therapy in the previous two centuries; however, the availability of opioids, coadjuvants, and newer anesthetic techniques has essentially replaced such procedures (65). The indication of these techniques is restricted to anecdotal reports nowadays.

#### Percutaneous Cervical Cordotomy

This procedure consists of creating a lesion to the lateral spinothalamic tract. The purpose is to disrupt the pain transmission carried from the contralateral side, as the spinothalamic tract carries pain, temperature, and some tactile information. The lesion is usually done percutaneously through the C1–C2 level (66). This procedure has been shown to be most effective in patients with confined unilateral nociceptive pain, such as in the case of mesothelioma (67) or other malignant invasions of the chest wall.

The complications involved are substantial with 3% mortality, up to 11% motor weakness, and others such as respiratory, postcordotomy hypotension, bladder dysfunction, sexual dysfunction, and dysethesia (68).

#### Intracerebroventricular Opioid Delivery

Intracerebroventricular opioids are useful for intractable pain when other simpler techniques have failed. It consists of delivering opioids *via* a ventricular catheter attached to a subcutaneous storage (69).

#### Cingulotomy

This procedure refers to lesioning of the anterior cingulate cortex, which is a component of the limbic system that affects a wide array of functions involving behavior, emotions, and others. It is indicated in cancer pain patients with significant emotional distress. A case report from 2014 described bilateral anterior cingulotomy effectively relieved both pain and dyspnea for a patient with malignant mesothelioma (70).

### Procedures for Localized Painful Bone Metastases

#### Cement Augmentation Techniques

The diagnosis and management of clinically relevant bone fractures are based on a clinical examination indicating pain localized to the level of the fracture along with confirmatory imaging studies (71). Cementoplasty refers to a technique where cement is delivered percutaneously to the spinal bones or other weight-bearing bones for stability purposes. It broadly includes procedures like vertebroplasty, kyphoplasty, sacroplasty, and osteoplasty (72).

#### Vertebroplasty and Kyphoplasty

Untreated vertebral compression fractures can result in a spinal cord compression with irreversible neurological symptoms and paraplegia (73, 74). Pain severity or the medications used to control pain can cause considerable functional impairment, significantly limiting patients’ mobility and ability to carry out day-to-day activities (75).

Vertebral augmentation techniques—vertebroplasty or kyphoplasty—are often done at an outpatient setting, at which image-guided injection of bone cement (methyl methacrylate) is injected into a collapsed vertebral body. This approach may be valuable for patients when pain is unresponsive to conservative treatments and no other options like RT are available, and for patients whose pain causes poor functional status thus limiting their life expectancy. Compared to non-surgical management, kyphoplasty was found to be an effective and safe treatment that rapidly reduced pain and improved function. A recently published systematic review including 111 clinical reports with 4,235 patients evaluated vertebral augmentation (vertebroplasty or kyphoplasty) for cancer-related vertebral compression fractures. Researchers found these two procedures to significantly and rapidly reduced pain intensity as well as significantly decrease the need for opioid pain medication, and functional disabilities related to back and neck pain (76). Beyond the contraindications mentioned above for invasive procedures, additional contraindications for these procedures include epidural disease, a fracture with new neurological impairment attributed to it, and fractured vertebra with a burst element penetrating the spinal canal (77).

#### Osteoplasty

It is the percutaneous injection of bone cement into painful bone metastases at extraspinal regions. Two retrospective studies, comprising a total of 76 patients, evaluated osteoplasty under CT or fluoroscopy found this technique effective and valuable as a method for reduction of pain and improvement of patients’ quality of life (78, 79). In particular, for patients with lung cancer metastatic to the bones, a large retrospective series demonstrated vertebroplasty and cementoplasty to be effective and safe as a means to decrease pain and enhance mobility in patients with vertebral and extra spinal metastases (80).

#### RF Ablation and Cryoablation of Painful Bone Metastases

Several new ablation treatment strategies have been reported to be effective over the last two decades. These treatments consist of image-guided (CT, fluoroscopy) destruction of soft tissues or bone tumors (either primary or metastatic). Among these techniques, RF ablation is the most studied and frequently used modality, but cryoablation, laser ablation, and microwave ablation have all been also reported. The pain treated with these techniques should be limited to one or two sites, and patients with numerous painful tumors should be treated systemically. Ablative therapy tends to be most effective in soft-tissue tumors and bone tumors with dominant osteolytic component.

#### RF Ablation

High-frequency, alternating current is passed to an adjacent tissue *via* a needle electrode and results in heating of the tissue, denaturation of proteins, and cell death. It is usually performed with local anesthesia or under moderate sedation. Careful consideration of the regional anatomy should be carefully assessed and considered. As anatomy is frequently disturbed in these patients, the ablation zone should not be extended to less than 1 cm of critical structures such as the bowel, urinary bladder, or spine (81). Two multicenter clinical trials conducted on a large set of patients with a wide range of solid malignancies (the majority were lung, colon, and renal metastases) confirmed that RF ablation as a means to decrease pain due to bone metastatic disease is safe and well tolerated by patients (82, 83).

#### Percutaneous Cryoablation

This method uses room temperature-pressurized argon and helium gasses for tissue freezing and warming, respectively. The cryoprobes are placed into the tumor using CT/fluoroscopy for tumors within bones or deep in the pelvis. The passage of gas through the probe results in rapid cooling that reaches −100°C within a few seconds, forming a low-attenuation ice ball that is readily visible with the CT imaging (or MRI). Tissue destruction is complete at −20 to −40°C, approximately 3- to 5-mm deep to the visible edge of the ice ball. A synchronous use of multiple cryoprobes can be done to allow for a complete coverage of the tumor and its immediate surroundings. The method is less studied than RFA, but case series and reports have established its efficacy. Its main advantage over RF is its ability to be readily visualized intra-procedurally with intermittent non-enhanced CT or MR imaging (84).

## Integrating Interventional Pain Treatment into an Oncology Practice

Multidisciplinary symptom management results in positive outcomes described in terms of significant relief of cancer pain and other cancer-related symptoms like fatigue, depression, anxiety, and drowsiness. It also impacts positively on patients’ disability and eventually on opioid reduction (85).

Traditionally, interventional treatments have been regarded as a last resort to relieving cancer pain in those patients where conventional drug therapies have failed. The term “fourth step of the WHO ladder” was coined with views of placing interventional cancer pain within the well-known WHO three steps clinical algorithm (86). Major efforts are being conducted to prove that interventional pain management indicated at early stages of the disease or before the pain becomes unmanageable with drugs may be a better option. Potential benefits of early blocks include better health status and enhanced performance to face the disease and its treatment and avoiding or delaying opioid escalation to manage pain. Rather than a fourth step of the ladder, interventional cancer pain approaches should be regarded as a handrail accompanying all the three steps of the WHO ladder.

Interaction between different clinical specialties may be challenging if their mutual approaches are poorly understood or perceived ineffective and/or dangerous. In the case of interventional cancer pain management, this is more challenging since the outcomes can seldom be presented in terms of evidence-based medicine.

Oncologist must identify those patients whose pain is inadequately controlled and ask themselves if an interventional approach may be indicated. With progressive learning, the indications and contraindications become clearer, and the cases are referred in a timelier and more appropriate fashion. Interventional pain clinicians must identify, in turn, the potential implications of their techniques on the patient’s status like, for example, the risk of bleeding when anticoagulated or receiving chemotherapy, the anatomical alterations a tumor may cause when attempting to target a specific nerve structure, or the changes in analgesic therapy necessary to apply after a successful nerve block or neurolysis. A fluid and bidirectional communication is key to integrate successful analgesic strategies into the oncology care.

## Summary

Interventional cancer pain approaches can provide valuable help to treating oncologist in cases of lung cancer with pain that is not satisfactorily relieved with conventional medical management. The indications and contraindications, the goals of treatment, the limitation of the technique, and the post-procedure care are necessary elements to be discussed between clinicians involved and the patient and their caregivers. Because the available scientific evidences are sparse, at present, interventional cancer pain remains an optional alternative rather than a natural indication. Only those teams integrating a specialist in interventional cancer pain may offer these options to selected cases presenting with challenging cancer pain syndromes.

## Author Contributions

JP has collected the contribution from the other co-signing authors, edited and formatted the text, and prepared the final manuscript for submission. UH and ME contributed to the preparation of the manuscript and collected data from the medical literature to prepare the review.

## Conflict of Interest Statement

The authors declare that the research was conducted in the absence of any commercial or financial relationships that could be construed as a potential conflict of interest.

## References

[1] Van den Beuken-van Everdingen MH Hochstenbach LM Joosten EA Tjan-Heijnen VC Janssen DJ. Update on prevalence of pain in patients with cancer: systematic review and meta-analysis. J Pain Symptom Manage (2016) 51:1070–90. 10.1016/j.jpainsymman.2015.12.340 27112310

[2] Aldarouish M Wang C Trends and advances in tumor immunology and lung cancer immunotherapy. J Exp Clin Cancer Res (2016) 35:157 10.1186/s13046-016-0439-3 27686848 PMC5043622

[3] Pergolizzi JV Gharibo C Ho KY Treatment considerations for cancer pain: a global perspective. Pain Pract (2015) 15:778–92. 10.1111/papr.12253 25469726

[4] World Health Organization. Cancer Pain Relief with a Guide to Opioid Availability. 2nd ed Geneva, Switzerland: World Health Organization (1996).

[5] Meuser T Pietruck C Radbruch L Stute P Lehmann KA Grond S. Symptoms during cancer pain treatment following WHO-guidelines: a longitudinal follow-up study of symptom prevalence, severity and etiology. Pain (2001) 93:247–57. 10.1016/S0304-3959(01)00324-4 11514084

[6] Vissers KC Besse K Wagemans M Zuurmond W Giezeman MJ Lataster A Evidence-based interventional pain medicine according to clinical diagnoses: pain in patients with cancer. Pain Pract (2011) 11:453–75. 10.1111/j.1533-2500.2011.00473.x 21679293

[7] Mercadante S Vitrano V Pain in patients with lung cancer: pathophysiology and treatment. Lung Cancer (2010) 68:10–5. 10.1016/j.lungcan.2009.11.004 20007003

[8] Simmons CP Macleod N Laird BJ. Clinical management of pain in advanced lung cancer. Clin Med Insights Oncol (2012) 6:331–46. 10.4137/CMO.S8360 23115483 PMC3474460

[9] Chute CG Greenberg ER Baron J Korson R Baker J Yates J. Presenting conditions of 1539 population-based lung cancer patients by cell type and stage in New Hampshire and Vermont. Cancer (1985) 56:2107–11. 10.1002/1097-0142(19851015)56:8<2107::AID-CNCR2820560837>3.0.CO;2-T 2992757

[10] Warren WH Chest wall involvement including Pancoast tumours. In: Pass HI Mitchell JB Johnson DH, editors. Lung Cancer Principles and Practise. Philadelphia: Lippincott Williams & Wilkins (2000). p. 716–29.

[11] Parker C Neville E Lung cancer: management of malignant mesothelioma. Thorax (2003) 58:809–13. 10.1136/thorax.58.9.809 12947146 PMC1746789

[12] Tubiana-Hulin M Incidence, prevalence and distribution of bone metastases. Bone (1991) 12:S9–10. 10.1016/8756-3282(91)90059-R 1954049

[13] Sirmali M Türüt H Topçu S Gülhan E Yazici U Kaya S A comprehensive analysis of traumatic rib fractures: morbidity, mortality and management. Eur J Cardiothorac Surg (2003) 24:133–8. 10.1016/S1010-7940(03)00256-2 12853057

[14] Zarogoulidis K Porpodis K Domvri K Eleftheriadou E Ioannidou D Zarogoulidis P. Diagnosing and treating pancoast tumors. Expert Rev Respir Med (2016) 10(12):1255–8. 10.1080/17476348.2017.1246964 27786592

[15] Mercadante S Neoplasm-induced pain. In: Gilman S, editor. Neurobiology of Disease. San Diego, CA: Elsevier (2007). p. 1007–20.

[16] Kehlet H Jensen TS Woolf CJ. Persistent postsurgical pain: risk factors and prevention. Lancet (2006) 367:1618–25. 10.1016/S0140-6736(06)68700-X 16698416

[17] Merskey H Bogduk H Classification of chronic pain. 2nd ed In: Merskey H Bogduk H, editors. Descriptions of Chronic Pain Syndromes and Definitions of Pain Terms. Seattle, WA: IASP Press (1994). p. 143–4.

[18] Hamada H Moriwaki K Shiroyama K Tanaka H Kawamoto M Yuge O. Myofascial pain in patients with postthoracotomy pain syndrome. Reg Anesth Pain Med (2000) 25:302–5. 10.1016/S1098-7339(00)90016-6 10834788

[19] Rusthoven JJ Ahlgren P Elhakim T Pinfold P Reid J Stewart L Varicella-zoster infection in adult cancer patients. A population study. Arch Intern Med (1988) 148:1561–6. 10.1001/archinte.148.7.1561 3382302

[20] Galer BS Portenoy RK. Acute herpetic and postherpetic neuralgia: clinical features and management. Mt Sinai J Med (1991) 58:257–66. 1875964

[21] Van Wijck AJ Wallace M Mekhail N van Kleef M. Evidence-based interventional pain medicine according to clinical diagnoses. 17. Herpes zoster and post-herpetic neuralgia. Pain Pract (2011) 11:88–97. 10.1111/j.1533-2500.2010.00428.x 21114617

[22] Nielsen OS Munro AJ Tannock IF. Bone metastases: pathophysiology and management policy. J Clin Oncol (1991) 9:509–24. 1705581 10.1200/JCO.1991.9.3.509

[23] Coleman RE Skeletal complications of malignancy. Cancer (1997) 80:1588–94. 9362426 10.1002/(sici)1097-0142(19971015)80:8+<1588::aid-cncr9>3.3.co;2-z

[24] Mercadante S Malignant bone pain: pathophysiology and treatment. Pain (1997) 69:1–18. 10.1016/S0304-3959(96)03267-8 9060007

[25] Sciubba DM Petteys RJ Dekutoski MB Fisher CG Fehlings MG Ondra SL Diagnosis and management of metastatic spine disease, a review. J Neurosurg Spine (2010) 13:94–108. 10.3171/2010.3.SPINE09202 20594024

[26] Tong D Gillick L Hendrickson FR. The palliation of symptomatic osseous metastases: final results of the study by the Radiation Therapy Oncology Group. Cancer (1982) 50:893–9. 10.1002/1097-0142(19820901)50:5<893::AID-CNCR2820500515>3.0.CO;2-Y 6178497

[27] Gaze MN Kelly CG Kerr GR Cull A Cowie VJ Gregor A Pain relief and quality of life following radiotherapy for bone metastases: a randomised trial of two fractionation schedules. Radiother Oncol (1997) 45:109–16. 10.1016/S0167-8140(97)00101-1 9423999

[28] Porta-Sales J Garzón-Rodríguez C Llorens-Torromé S Brunelli C Pigni A Caraceni A Evidence on the analgesic role of bisphosphonates and denosumab in the treatment of pain due to bone metastases: a systematic review within the European Association for Palliative Care guidelines project. Palliat Med (2017) 31:5–25. 10.1177/0269216316639793 27006430

[29] Twycross RG Fairfield S. Pain in far-advanced cancer. Pain (1982) 14:303–10. 10.1016/0304-3959(82)90137-3 6218464

[30] Torres Lacomba M Mayoral del Moral O Coperias Zazo JL Gerwin RD Goñí AZ. Incidence of myofascial pain syndrome in breast cancer surgery: a prospective study. Clin J Pain (2010) 26:320–5. 10.1097/AJP.0b013e3181c4904a 20393267

[31] Bennett R. Myofascial pain syndromes and their evaluation. Best Pract Res Clin Rheumatol (2007) 21:427–45. 10.1016/j.berh.2007.02.014 17602992

[32] Scott NA Guo B Barton PM Gerwin RD. Trigger point injections for chronic non-malignant musculoskeletal pain: a systematic review. Pain Med (2009) 10:54–69. 10.1111/j.1526-4637.2008.00526.x 18992040

[33] Ripamonti CI Bossi P Santini D Fallon M. Pain related to cancer treatments and diagnostic procedures: a no man’s land? Ann Oncol (2014) 25:1097–106. 10.1093/annonc/mdu011 24625453

[34] Ewertz M Qvortrup C Eckhoff L. Chemotherapy-induced peripheral neuropathy in patients treated with taxanes and platinum derivatives. Acta Oncol (2015) 54:587–91. 10.3109/0284186X.2014.995775 25751757

[35] Hird A Chow E Zhang L Wong R Wu J Sinclair E Determining the incidence of pain flare following palliative radiotherapy for symptomatic bone metastases: results from three Canadian cancer centers. Int J Radiat Oncol Biol Phys (2009) 75:193–7. 10.1016/j.ijrobp.2008.10.044 19167840

[36] Stubblefield MD. Radiation fibrosis syndrome: neuromuscular and musculoskeletal complications in cancer survivors. PM R (2011) 3:1041–4. 10.1016/j.pmrj.2011.08.535 22108231

[37] Dunlap NE Cai J Biedermann GB Yang W Benedict SH Sheng K Chest wall volume receiving >30 Gy predicts risk of severe pain and/or rib fracture after lung stereotactic body radiotherapy. Int J Radiat Oncol Biol Phys (2010) 76:796–801. 10.1016/j.ijrobp.2009.02.027 19427740

[38] Forquer JA Fakiris AJ Timmerman RD Lo SS Perkins SM McGarry RC Brachial plexopathy from stereotactic body radiotherapy in early-stage NSCLC: dose-limiting toxicity in apical tumor sites. Radiother Oncol (2009) 93:408–13. 10.1016/j.radonc.2009.04.018 19454366

[39] Sindt JE Brogan SE. Interventional treatments of cancer pain. Anesthesiol Clin (2016) 34:317–39. 10.1016/j.anclin.2016.01.004 27208713

[40] Simpson KA Interventional techniques for pain management in palliative care. Medicine (2011) 39:645–7. 10.1016/j.mpmed.2011.08.011

[41] Gulati A Shah R Puttanniah V Hung JC Malhotra V. A retrospective review and treatment paradigm of interventional therapies for patients suffering from intractable thoracic chest wall pain in the oncologic population. Pain Med (2015) 16:802–10. 10.1111/pme.12558 25236160

[42] Wong FC Lee TW Yuen KK Lo SH Sze WK Tung SY. Intercostal nerve blockade for cancer pain: effectiveness and selection of patients. Hong Kong Med J (2007) 13:266–70. 17664531

[43] Axelsson K Gupta A. Local anaesthetic adjuvants: neuraxial versus peripheral nerve block. Curr Opin Anaesthesiol (2009) 22:649–54. 10.1097/ACO.0b013e32832ee847 19593120

[44] Matchett G. Intercostal nerve block and neurolysis for intractable cancer pain. J Pain Palliat Care Pharmacother (2016) 30:114–7. 10.3109/15360288.2016.1167804 27092398

[45] Ahmed A Bhatnagar S Khurana D Joshi S Thulkar S. Ultrasound-guided radiofrequency treatment of intercostal nerves for the prevention of incidental pain arising due to rib metastasis: a prospective study. Am J Hosp Palliat Care (2015) 34:115–24. 10.1177/1049909115617933 26656033

[46] Gulati A Loh J Gutta NB Ezell PC Monette S Erinjeri JP Novel use of noninvasive high-intensity focused ultrasonography for intercostal nerve neurolysis in a swine model. Reg Anesth Pain Med (2014) 39:26–30. 10.1097/AAP.0000000000000028 24317231 PMC9382831

[47] Ju H Feng Y Yang BX Wang J. Comparison of epidural analgesia and intercostal nerve cryoanalgesia for post-thoracotomy pain control. Eur J Pain (2008) 12:378–84. 10.1016/j.ejpain.2007.07.011 17870625

[48] Cohen SP Sireci A Wu CL Larkin TM Williams KA Hurley RW. Pulsed radiofrequency of the dorsal root ganglia is superior to pharmacotherapy or pulsed radiofrequency of the intercostal nerves in the treatment of chronic postsurgical thoracic pain. Pain Physician (2006) 9:227–35. 16886031

[49] Antila H Kirvelä O. Neurolytic thoracic paravertebral block in cancer pain. A clinical report. Acta Anaesthesiol Scand (1998) 42:581–5. 10.1111/j.1399-6576.1998.tb05170.x 9605376

[50] Vranken JH Zuurmond WW de Lange JJ. Continuous brachial plexus block as treatment for the Pancoast syndrome. Clin J Pain (2000) 16:327–33. 10.1097/00002508-200012000-00009 11153789

[51] Mullin V Brachial plexus block with phenol for painful arm associated with Pancoast’s syndrome. Anesthesiology (1980) 53:431–3. 7425379

[52] Gorlin AW Rosenfeld DM Maloney J Wie CS McGarvey J Trentman TL. Survey of pain specialists regarding conversion of high-dose intravenous to neuraxial opioids. J Pain Res (2016) 21:693–700. 10.2147/JPR.S113216 27703394 PMC5036565

[53] Wagemans MF Zuurmond WW de Lange JJ. Long-term spinal opioid therapy in terminally ill cancer pain patients. Oncologist (1997) 2:70–5. 10388033

[54] Hogan Q Haddox JD Abram S Weissman D Taylor ML Janjan N. Epidural opiates and local anesthetics for the management of cancer pain. Pain (1991) 46:271–9. 10.1016/0304-3959(91)90110-J 1758711

[55] Deer TR Smith HS Burton AW Pope JE Doleys DM Levy RM Comprehensive consensus based guidelines on intrathecal drug delivery systems in the treatment of pain caused by cancer pain. Pain Physician (2011) 14:283–312. 21587338

[56] Nitescu P Appelgren L Linder LE Sjöberg M Hultman E Curelaru I. Epidural versus intrathecal morphine-bupivacaine: assessment of consecutive treatments in advanced cancer pain. J Pain Symptom Manage (1990) 95:18–26. 10.1016/S0885-3924(05)80005-3 2324557

[57] Smith TJ Staats PS Deer T Stearns LJ Rauck RL Boortz-Marx RL Implantable drug delivery systems study group. Randomized clinical trial of an implantable drug delivery system compared with comprehensive medical management for refractory cancer pain: impact on pain, drug-related toxicity, and survival. J Clin Oncol (2002) 20:4040–9. 10.1200/JCO.2002.02.118 12351602

[58] Candido K Stevens RA. Intrathecal neurolytic blocks for the relief of cancer pain. Best Pract Res Clin Anaesthesiol (2003) 17:408–28. 10.1016/S1521-6896(03)00016-8 14529011

[59] Patt RB Reddy S. Spinal neurolysis for cancer pain: indications and recent results. Ann Acad Med Singapore (1994) 23:216–20. 8080224

[60] El-Sayed GG. A new catheter technique for thoracic subarachnoid neurolysis in advanced lung cancer patients. Pain Pract (2007) 7:27–30. 10.1111/j.1533-2500.2007.00106.x 17305675

[61] Cameron T. Safety and efficacy of spinal cord stimulation for the treatment of chronic pain: a 20-year literature review. J Neurosurg (2004) 100:254–67. 15029914 10.3171/spi.2004.100.3.0254

[62] Peng L Min S Zejun Z Wei K Bennett MI. Spinal cord stimulation for cancer-related pain in adults. Cochrane Database Syst Rev (2015). 10.1002/14651858.CD009389.pub3 26121600 PMC6464643

[63] Yakovlev AE Resch BE Karasev SA. Treatment of cancer-related chest wall pain using spinal cord stimulation. Am J Hosp Palliat Care (2010) 27:552–6. 10.1177/1049909110373240 20555037

[64] Cata JP Cordella JV Burton AW Hassenbusch SJ Weng HR Dougherty PM. Spinal cord stimulation relieves chemotherapy-induced pain: a clinical case report. J Pain Symptom Manage (2004) 27:72–8. 10.1016/j.jpainsymman.2003.05.007 14711471

[65] Raslan AM Cetas JS McCartney S Burchiel KJ. Destructive procedures for control of cancer pain: the case for cordotomy. J Neurosurg (2011) 114:155–70. 10.3171/2010.6.JNS10119 20690810

[66] Kanpolat Y Savas A Ucar T Torun F Percutaneous selective cordotomy for treatment of intractable pain in patients with malignant pleural mesothelioma. Acta Neurochir (Wien) (2002) 144:595–9. 10.1007/s00701-002-0945-2 12111493

[67] DeFrance BD Lewis RA Sharma ML Poolman M. Cordotomy in mesothelioma-related pain: a systematic review. BMJ Support Palliat Care (2014) 4:19–29. 10.1136/bmjspcare-2013-000508 24644767

[68] Raslan AM. Percutaneous computed tomography-guided radiofrequency ablation of upper spinal cord pain pathways for cancer-related pain. Neurosurgery (2008) 62:226–33. 10.1227/01.neu.0000317397.16089.f5 18424990

[69] Lazorthes YR Sallerin BA Verdié JC. Intracerebroventricular administration of morphine for control of irreducible cancer pain. Neurosurgery (1995) 37:422–8. 10.1097/00006123-199509000-00009 7501106

[70] Pereira EA Paranathala M Hyam JA Green AL Aziz TZ. Anterior cingulotomy improves malignant mesothelioma pain and dyspnoea. Br J Neurosurg (2014) 28:471–4. 10.3109/02688697.2013.857006 24199940

[71] Jakobs TF Trumm C Reiser M Hoffmann RT. Percutaneous vertebroplasty in tumoral osteolysis. Eur Radiol (2007) 17:2166–75. 10.1007/s00330-007-0581-6 17277947

[72] Katsanos K Sabharwal T Adam A. Percutaneous cementoplasty. Semin Intervent Radiol (2010) 27:137–47. 10.1055/s-0030-1253512 21629403 PMC3036515

[73] Schiff D O’Neill BP Suman VJ. Spinal epidural metastasis as the initial manifestation of malignancy: clinical features and diagnostic approach. Neurology (1997) 49:452–6. 10.1212/WNL.49.2.452 9270576

[74] Zaikova O Giercksky KE Fossa SD Kvaloy S Johannesen TB Skjeldal S. A population-based study of spinal metastatic disease in South-East Norway. Clin Oncol (R Coll Radiol) (2009) 21(10):753–9. 10.1016/j.clon.2009.09.023 19850454

[75] Costa L Badia X Chow E Lipton A Wardley A Impact of skeletal complications on patients’ quality of life, mobility, and functional independence. Support Care Cancer (2008) 16:879–89. 10.1007/s00520-008-0496-z 18392862

[76] Berenson J Pflugmacher R Jarzem P Zonder J Schechtman K Tillman JB Balloon kyphoplasty versus non-surgical fracture management for treatment of painful vertebral body compression fractures in patients with cancer: a multicentre, randomised controlled trial. Lancet Oncol (2011) 12:225–35. 10.1016/S1470-2045(11)70008-0 21333599

[77] Health Quality Ontario. Vertebral augmentation involving vertebroplasty or kyphoplasty for cancer-related vertebral compression fractures: a systematic review. Ont Health Technol Assess Ser (2016) 16:1–202. PMC4902848 27298655

[78] Maccauro G Liuzza F Scaramuzzo L Milani A Muratori F Rossi B Percutaneous acetabuloplasty for metastatic acetabular lesions. BMC Musculoskelet Disord (2008) 9:66. 10.1186/1471-2474-9-66 18457594 PMC2390555

[79] Sun G Jin P Liu XW Li M Li L. Cementoplasty for managing painful bone metastases outside the spine. Eur Radiol (2014) 24:731–7. 10.1007/s00330-013-3071-z 24272227

[80] Wang Z Zhen Y Wu C Li H Yang Y Shen Z CT fluoroscopy-guided percutaneous osteoplasty for the treatment of osteolytic lung cancer bone metastases to the spine and pelvis. J Vasc Interv Radiol (2012) 23:1135–42. 10.1016/j.jvir.2012.06.007 22920977

[81] Sabharwal T Katsanos K Buy X Gangi A. Image-guided ablation therapy of bone tumors. Semin Ultrasound CT MR (2009) 30:78–90. 10.1053/j.sult.2008.12.003 19358439

[82] Goetz MP Callstrom MR Charboneau JW Farrell MA Maus TP Welch TJ Percutaneous image-guided radiofrequency ablation of painful metastases involving bone: a multicenter study. J Clin Oncol (2004) 22:300–6. 10.1200/JCO.2004.03.097 14722039

[83] Dupuy DE Liu D Hartfeil D Hanna L Blume JD Ahrar K Percutaneous radiofrequency ablation of painful osseous metastases: a multicenter American College of Radiology Imaging Network trial. Cancer (2010) 116:989–97. 10.1002/cncr.24837 20041484 PMC2819592

[84] Callstrom MR Dupuy DE Solomon SB Beres RA Littrup PJ Davis KW Percutaneous image-guided cryoablation of painful metastases involving bone: multicenter trial. Cancer (2013) 119:1033–41. 10.1002/cncr.27793 23065947 PMC5757505

[85] Perez J Olivier S Rampakakis E Borod M Shir Y. The McGill University Health Centre cancer pain clinic: a retrospective analysis of an interdisciplinary approach to cancer pain management. Pain Res Manag (2016) 2016:2157950. 10.1155/2016/2157950 27445602 PMC4904607

[86] Miguel R. Interventional treatment of cancer pain: the fourth step in the World Health Organization analgesic ladder? Cancer Control (2000) 7:149–56. 10783819 10.1177/107327480000700205

